# Evolution of Intestinal Gases and Fecal Short-Chain Fatty Acids Produced *in vitro* by Preterm Infant Gut Microbiota During the First 4 Weeks of Life

**DOI:** 10.3389/fped.2021.726193

**Published:** 2021-09-27

**Authors:** Xuefang Wang, Juan Li, Na Li, Kunyu Guan, Di Yin, Huating Zhang, Guodong Ding, Yong Hu

**Affiliations:** ^1^Department of Neonatology, Shanghai Children's Hospital, Shanghai Jiao Tong University, Shanghai, China; ^2^Department of Respiratory, Shanghai Children's Hospital, Shanghai Jiao Tong University, Shanghai, China

**Keywords:** intestinal gas, fecal short-chain fatty acid, carbon dioxide, hydrogen, methane, hydrogen sulfide

## Abstract

**Background:** The production of intestinal gases and fecal short-chain fatty acids (SCFAs) by infant gut microbiota may have a significant impact on their health, but information about the composition and volume of intestinal gases and SCFA profiles in preterm infants is scarce.

**Objective:** This study examined the change of the composition and volume of intestinal gases and SCFA profiles produced by preterm infant gut microbiota *in vitro* during the first 4 weeks of life.

**Methods:** Fecal samples were obtained at five time points (within 3 days, 1 week, 2 weeks, 3 weeks, and 4 weeks) from 19 preterm infants hospitalized in the neonatal intensive care unit (NICU) of Shanghai Children's Hospital, Shanghai Jiao Tong University between May and July 2020. These samples were initially inoculated into four different media containing lactose (LAT), fructooligosaccharide (FOS), 2′-fucosyllactose (FL-2), and galactooligosaccharide (GOS) and thereafter fermented for 24 h under conditions mimicking those of the large intestine at 37.8°C under anaerobic conditions. The volume of total intestinal gases and the concentrations of individual carbon dioxide (CO_2_), hydrogen (H_2_), methane (CH_4_), and hydrogen sulfide (H_2_S) were measured by a gas analyzer. The concentrations of total SCFAs, individual acetic acid, propanoic acid, butyric acid, isobutyric acid, pentanoic acid, and valeric acid were measured by gas chromatography (GC).

**Results:** The total volume of intestinal gases (ranging from 0.01 to 1.64 ml in medium with LAT; 0–1.42 ml with GOS; 0–0.91 ml with FOS; and 0–0.44 ml with FL-2) and the concentrations of CO_2_, H_2_, H_2_S, and all six fecal SCFAs increased with age (*p*-trends < 0.05). Among them, CO_2_ was usually the predominant intestinal gas, and acetic acid was usually the predominant SCFA. When stratified by birth weight (<1,500 and ≥1,500 g), gender, and delivery mode, the concentration of CO_2_ was more pronounced among infants whose weight was ≥1,500 g than among those whose weight was <1,500 g (*p*-trends < 0.05).

**Conclusions:** Our findings suggested that the intestinal gases and SCFAs produced by preterm infant gut microbiota *in vitro* increased with age during the first 4 weeks of life.

## Introduction

The abundant and diverse members of the human gut microbiota have intricate roles in maintaining health (1, 2). A growing number of studies have attempted to characterize the composition of the gut microbiota using rRNA and DNA technologies, while much less is known about the metabolism of the intestinal microbiota *in situ* and in real time. Their byproducts, including intestinal gases and short-chain fatty acids (SCFAs), may be unique biomarkers for specific gut microbiota (3–5). Measuring these byproducts may accelerate our understanding of the relationships among intestinal gases, SCFAs, the metabolic activity of the gut microbiota, and human health states.

Previous studies indicated that a significant portion of the intestinal gases are produced through fermentation in the gut instead of the human host. Intestinal gases mainly include carbon dioxide (CO_2_), hydrogen (H_2_), methane (CH_4_), and hydrogen sulfide (H_2_S) (3, 6). Michael et al. measured the volume and composition of intestinal gases from 11 healthy, young male subjects by means of an intestinal washout technique. They found that the bowel normally contains relatively small quantities of gas ranging from 30 to 200 ml, and nitrogen (N_2_) was usually the predominant gas, whereas oxygen (O_2_) was less (7). According to a study performed in 20 healthy individuals, the primary constituents of flatus are N_2_ (59%), H_2_ (20.9%), CO_2_ (9%), CH_4_ (7.2%), O_2_ (3.9%), and H_2_S (0.00028%) (8). H_2_ and CH_4_ can be detected using breath tests as markers of carbohydrate fermentation following a carbohydrate load or the occurrence of small intestinal bacterial overgrowth (SIBO) (9). Microbes colonize the neonatal gut immediately following birth (1, 10). The gut bacterial communities in adults are different from those in infants. The intestinal gases produced by gut microbiota are also different between them. The volume and composition of intestinal gases produced by the infant gut microbiota are unknown, especially in preterm infants.

SCFAs, important byproducts of the complex interplay between diet and the gut microbiota within the gut lumen environment, are regarded as key signaling molecules between the gut microbiome and host health states (11). It is well established that SCFAs play a profound role in mucosal maintenance and integrity, glucose homeostasis, lipid metabolism, and immune function (4). Previous analysis of fecal SCFAs showed their potential to be used to detect patients with colorectal cancer (CRC) and adenomatous polyposis (AP) as a noninvasive means (12). In infants, the reduced concentrations of acetic acid and propionic acid may be related to the risk of breast milk jaundice (BMJ) (13). In addition, fecal SCFAs may be biomarkers of cow's milk protein allergy (CMPA) (14). Until now, there has been a study reporting the average total fecal SCFA concentrations and fecal butyrate concentrations of eight healthy adults over 12 weeks (15). However, information on the change in fecal SCFAs from meconium to feces during the first month of life is scarce, particularly in relation to preterm infants.

Therefore, the objective of our work was to reveal the evolution of the production of intestinal gases and fecal SCFAs *in vitro* by preterm infant gut microbiota during the first month of life. In addition, we made the observations about the role of birth weight, gender, delivery mode, and type of feeding for intestinal gas production and SCFAs after preterm birth.

## Materials and Methods

### Participants and Sample Collection

This study included 19 preterm babies hospitalized in the neonatal intensive care unit (NICU) of Shanghai Children's Hospital, Shanghai Jiao Tong University (Shanghai, China) between May and July 2020. Written informed parental consent was obtained for each preterm infant before inclusion. To be eligible for enrollment, preterm infants needed to be born at a complete gestational age (GA) of <37 weeks and without obvious malformations or inherited or metabolic diseases.

Spontaneously evacuated meconium and fecal samples were collected from diapers by the medical staff into 10-ml stool containers. Meconium was collected within 3 days after birth, and fecal samples were collected weekly from preterm infants when they were in the hospital, immediately stored at 4°C, and transported within 12 h for further fermentation.

### Fermentation *in vitro*

Fermentation *in vitro* was carried out as previously described (16). The four types of culture media consisted of the following common compounds: peptone water, 5.0 g/L; tryptone, 5.0 g/L; yeast extract, 2.5 g/L; l-aminothiopropionic acid, 1 g/L; hemin, 2 ml/L; NaCl, 0.9 g/L; CaCl_2_·6H_2_O, 0.09 g/L; KH_2_PO_4_, 0.45 g/L; K_2_HPO_4_, 0.45 g/L; MgSO_4_·7H_2_O, 0.09 g/L; resazurin (1 g/L), 1 ml; and vitamin I solution (including biotin, 0.05 g/L; cobalamin, 0.05 g/L; *para*-aminobenzoic acid, 0.15 g/L; folic acid, 0. 25 g/L; and pyridoxamine, 0. 75 g/L), 200 μl/L. We added LAT, 8 g/L; galactooligosaccharide (GOS), 8 g/L; fructooligosaccharide (FOS), 8 g/L; and 2′-fucosyllactose (FL-2), 8 g/L as the sole carbon source in the four types of media. Each medium was inoculated with 0.5 ml of 10% fresh fecal slurry prepared with prereduced phosphate-buffered saline (PBS) buffer (150 mmol/L of NaCl, 10 mmol/L of Na_2_HPO_4_, and 20 mmol/L of NaH_2_PO_4_, pH 7.2–7.4). Then, they were fermented for 24 h under conditions mimicking those of the large intestine at 37.8°C under complete anaerobic conditions.

### Intestinal Gas Measurement

The gas produced from preterm infant intestinal flora fermentation was detected by a gas analyzer (HL-QT01), which consists of a gas sampler, valving module, vacuum generator, and gas detection chamber integrated with several gas sensors. The valving module was used to control the gas quantity introduced into the gas detection chamber with the help of the vacuum generator. The programmable working process control and the sensing results analysis were performed by the electronics mainboard with preset software. The detection steps were as follows: (1) the vacuum generator adjusted the gas detection chamber to a certain vacuum level through the valving module; (2) the gas entered the instrument from the sample bottle through the gas sampler, and the gas volume was adjusted through the valving module; (3) the gas entered into the gas detection chamber was measured by gas sensors; and (4) the measurement results were calculated by preset software to obtain the gas content and bacterial composition in the sample container.

We calibrated the gas analyzer by detecting the gas in the medium that was not inoculated with fecal slurry and ensuring that the detection result was zero.

### Short-Chain Fatty Acid Measurement

The concentrations of SCFAs were measured by gas chromatography (GC). The amounts of acetic acid, propanoic acid, butyric acid, isobutyric acid, pentanoic acid, and valeric acid in the culture media were measured by GC (Shimadzu, GC-2010 Plus, Kyoto, Japan) equipped with a DB-FFAP column (0.32 mm ^*^ 30 m ^*^ 0.5 mm) (Agilent Technologies, Santa Clara, CA, USA) using an H_2_ flame ionization detector.

### Statistical Analysis

Statistical analyses were conducted using SPSS 26.0 software (SPSS Inc., Chicago, IL, USA). Quantitative data are expressed as the mean and 95% confidence intervals (CIs) of the mean or, when they were not nonnormally distributed, as the median and interquartile range (IQR). When classified by age, the Kruskal–Wallis test was used for comparison between groups. When classified by sex, delivery mode, or weight, the Mann–Whitney *U* test was used for comparisons among groups. *p* < 0.05 was considered to indicate a statistically significant difference.

## Results

### Infant Characteristics

Nineteen preterm infants (12 males and seven females) enrolled in the study had a mean GA of 31.11 (95% CI: 30.01, 32.20) weeks and a mean birth weight of 1,385.53 (95% CI: 1,235.89, 1,535.17) g. Of these 19 infants, nine were delivered by cesarean section, and 10 infants were delivered vaginally. Six infants received mechanical ventilation. Eleven infants were both breastfed and formula-fed, seven infants were exclusively breastfed, and only one infant was exclusively formula-fed. All infants received antibacterial prophylaxis for at least the first 5 days of life. The sociodemographic characteristics of the infants are presented in Table 1.

**Table 1:** Demographic and clinical characteristics of the preterm infants in this study (*n* = 19).

**No**.	**Gestational age (week)**	**Birth weight (g)**	**Gender**	**Delivery mode**	**Apgar scores at 1, 5, (10) min**	**Type of feeding**	**Antibiotherapy (days)**	**Mechanical ventilation**
1	31.57	1,300	Male	Cesarean section	8–9	Non-pure breast feeding	15	No
2	30	1,430	Male	Vaginal	9–9	Non-pure breast feeding	9	No
3	32.43	1,460	Male	Cesarean section	8–9	Non-pure breast feeding	5	No
4	36.43	1,770	Male	Cesarean section	9–9	Pure breast feeding	8	No
5	29.86	1,485	Male	Vaginal	7–4–(8)	Non-pure breast feeding	5	No
6	32	1,595	Male	Cesarean section	10	Pure breast feeding	10	No
7	33.29	1,480	Female	Cesarean section	8–9	Pure breast feeding	7	No
8	33.14	1,645	Male	Cesarean section	9–9	Pure breast feeding	19	No
9	28.14	1,125	Male	Vaginal	8–9	Pure breast feeding	28	Yes
10	28.14	1,050	Female	Vaginal	5–7–(7)	Pure breast feeding	23	Yes
11	28.14	970	Male	Vaginal	3–6–(9)	Pure breast feeding	26	Yes
12	34.14	2,040	Male	Vaginal	8–9	Non-pure breast feeding	6	No
13	29.14	1,415	Female	Cesarean section	8–9	Non-pure breast feeding	13	No
14	31.43	985	Female	Cesarean section	8–8	Pure breast feeding	25	Yes
15	28.43	1,040	Male	Vaginal	8–9	Non-pure breast feeding	13	Yes
16	31.43	1,575	Female	Vaginal	8–9	Non-pure breast feeding	21	No
17	32.29	1,560	Male	Vaginal	8–9	Non-pure breast feeding	7	No
18	29.86	850	Female	Cesarean section	6–9	Pure breast feeding	28	Yes
19	31.14	1,550	Female	Vaginal	8–9	Non-pure breast feeding	6	No

### Intestinal Gases

**Figure 1:**
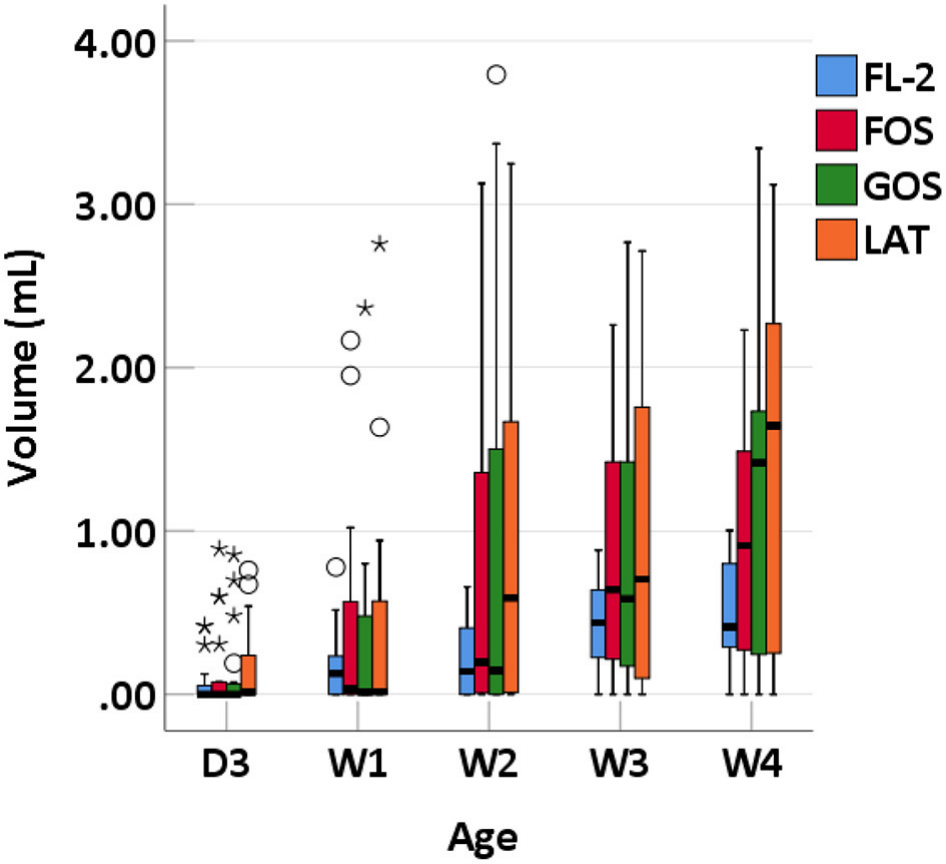
Total volume of intestinal gases of preterm infants of different ages. D3, 3 days after birth; W1, 1 week after birth; W2, 2 weeks after birth; W3, 3 weeks after birth; W4, 4 weeks after birth; LAT, lactose; FOS, fructooligosaccharide; FL-2, milk oligosaccharide; GOS, galactooligosaccharide; ^*^, extreme value; °, discrete value.

**Figure 2:**
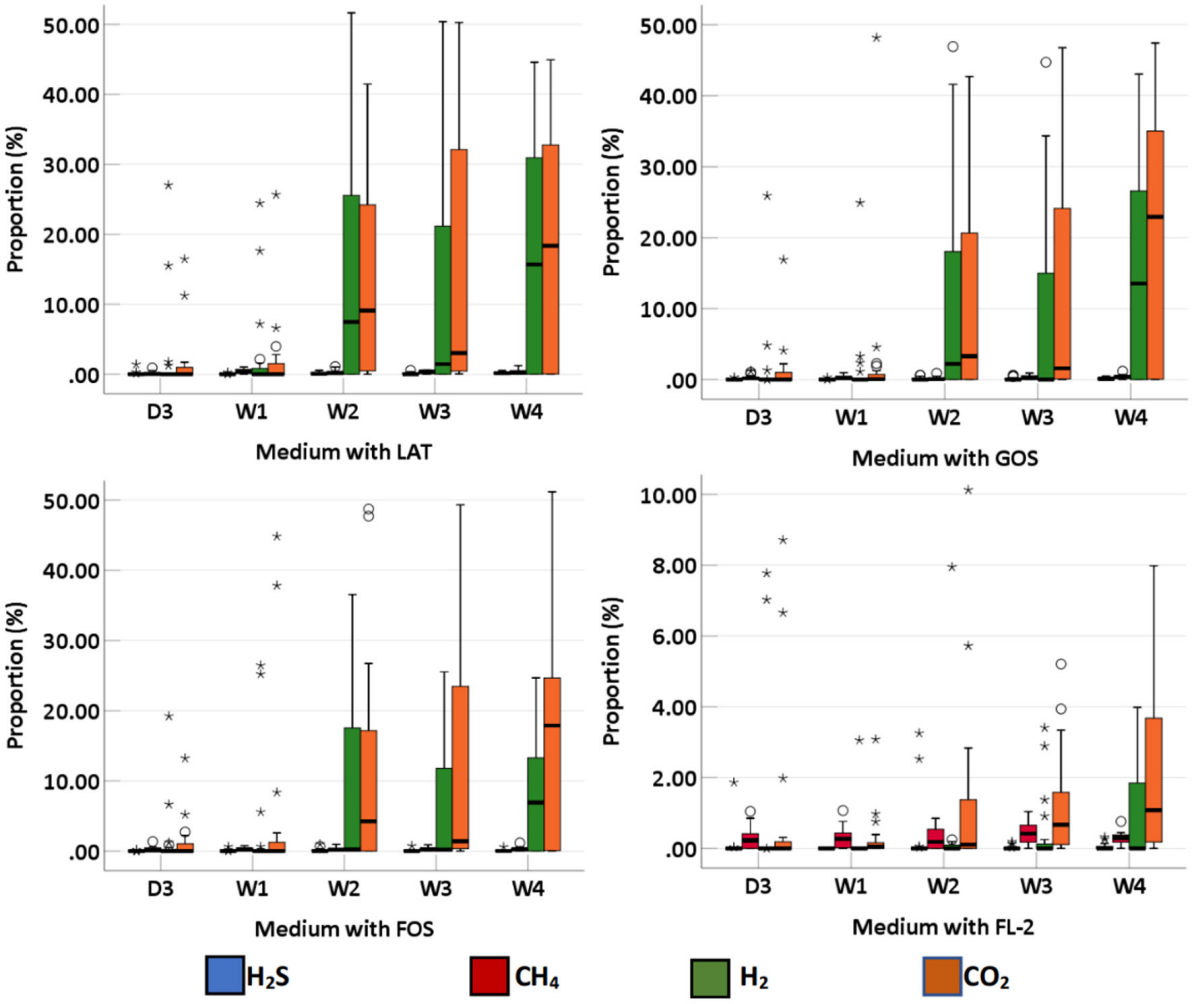
The proportion of intestinal gas composition (CO_2_, H_2_, CH_4_, and H_2_S) of preterm infants of different ages. D3, 3 days after birth; W1, 1 week after birth; W2, 2 weeks after birth; W3, 3 weeks after birth; W4, 4 weeks after birth; LAT, lactose; FOS, fructooligosaccharide; FL-2, milk oligosaccharide; GOS, galactooligosaccharide; ^*^, extreme value; °, discrete value.

**Figure 3:**
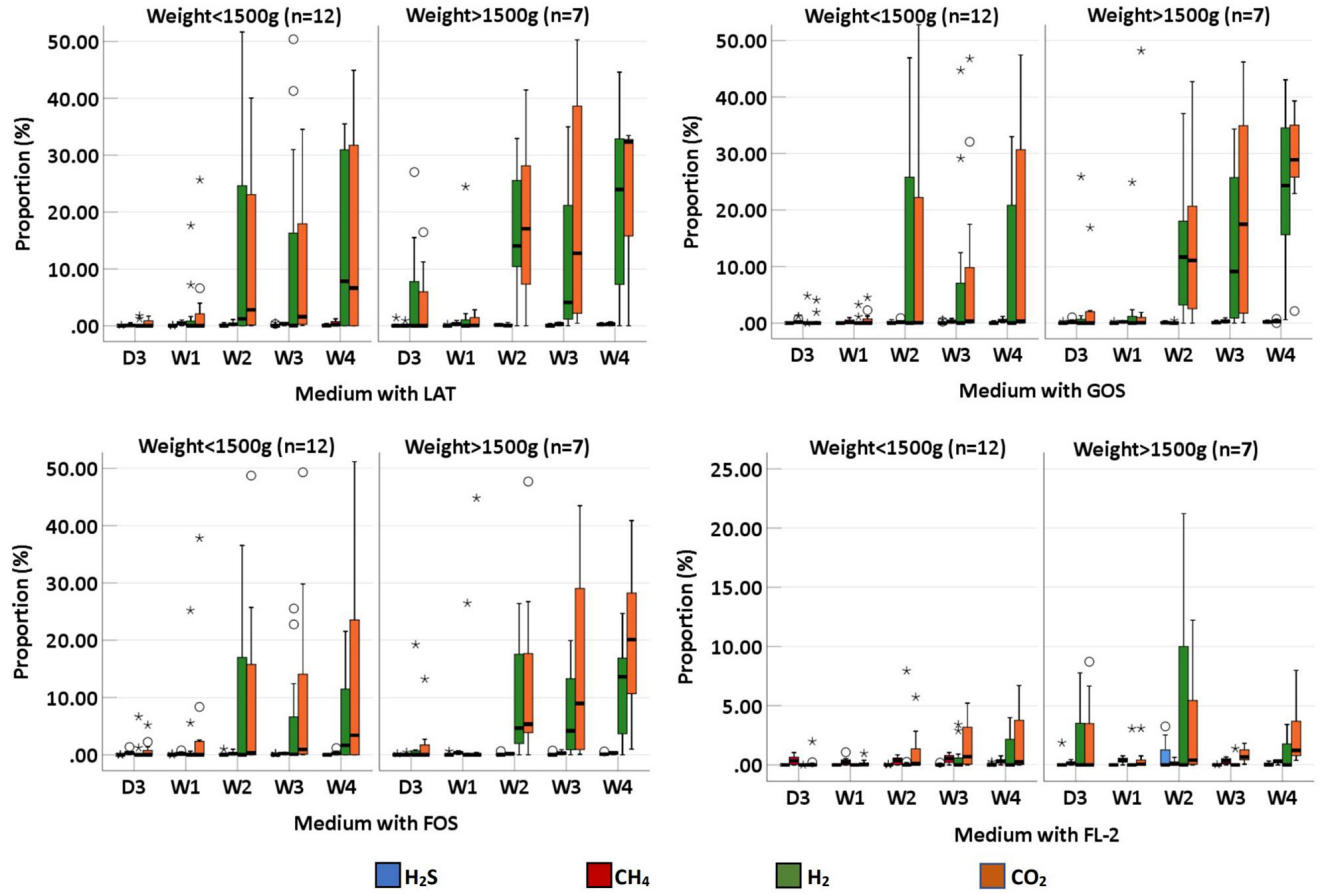
The proportion of intestinal gas composition (CO_2_, H_2_, CH_4_, and H_2_S) of preterm infants of different ages, stratified by weight (<1,500 and >1,500 g); D3, 3 days after birth; W1, 1 week after birth; W2, 2 weeks after birth; W3, 3 weeks after birth; W4, 4 weeks after birth; LAT, lactose; FOS, fructooligosaccharide; FL-2, milk oligosaccharide; GOS, galactooligosaccharide; ^*^, extreme value; °, discrete value.

We collected 95 stool specimens from 19 newborns and measured intestinal gases in 380 media. In medium with LAT, the total volume of intestinal gases produced by preterm infant gut microbiota was 0.01 (IQR: 0–0.31) ml within 3 days after birth (D3); 0.02 (IQR: 0–0.66) ml at 1 week after birth (W1); 0.60 (IQR: 0.01–1.72) ml at 2 weeks after birth (W2); 0.71 (IQR: 0.06–1.97) ml at 3 weeks after birth (W3); and 1.64 (IQR: 0.07–2.48) ml at 4 weeks after birth (W4). In medium with GOS, the total volume of intestinal gases was 0 (IQR: 0–0.75) ml within D3; 0.13 (IQR: 0–0.58) ml at W1; 0.15 (IQR: 0–1.71) ml at W2; 0.58 (IQR: 0.16–1.54) ml at W3; and 1.42 (IQR: 0.18–1.75) ml at W4. In medium with FOS, the total volume of intestinal gases was 0 (IQR: 0–0.77) ml within D3; 0.03 (IQR: 0–0.60) ml at W1; 0.20 (IQR: 0–1.71) ml at W2; 0.64 (IQR: 0.13–1.55) ml at W3; and 0.91 (IQR: 0.25–1.56) ml at W4. In medium with FL-2, the total volume of intestinal gases was 0 (IQR: 0–0.08) ml within D3; 0.13 (IQR: 0–0.24) ml at W1; 0.14 (IQR: 0–0.44) ml at W2; 0.44 (IQR: 0.20–0.55) ml at W3; and 0.41 (IQR: 0.25–0.80) ml at W4. The total volume of intestinal gases produced by preterm infant gut microbiota in the four media all increased with age (*p*-trends < 0.05) (Figure 1). The concentrations of CO_2_, H_2_, and H_2_S increased with age in the four media (*p*-trends < 0.05) (Figure 2). However, there was no difference in the concentration of CH_4_ among the five time points (p-trends > 0.05). CO_2_ and H_2_ are the two major intestinal gases. H_2_S was detectable in several samples (36/95). The detectable frequency of CH_4_ (75/95) was higher than that of H_2_S. When stratified by birth weight (<1,500 and ≥1,500 g), the concentration of CO_2_ was greater among infants whose birth weight was ≥1,500 g (*p*-trends < 0.05) (Figure 3). When stratified by gender, type of feeding, or delivery mode, differences were not found between the two groups (*p*-trends > 0.05).

### Short-Chain Fatty Acids

**Figure 4:**
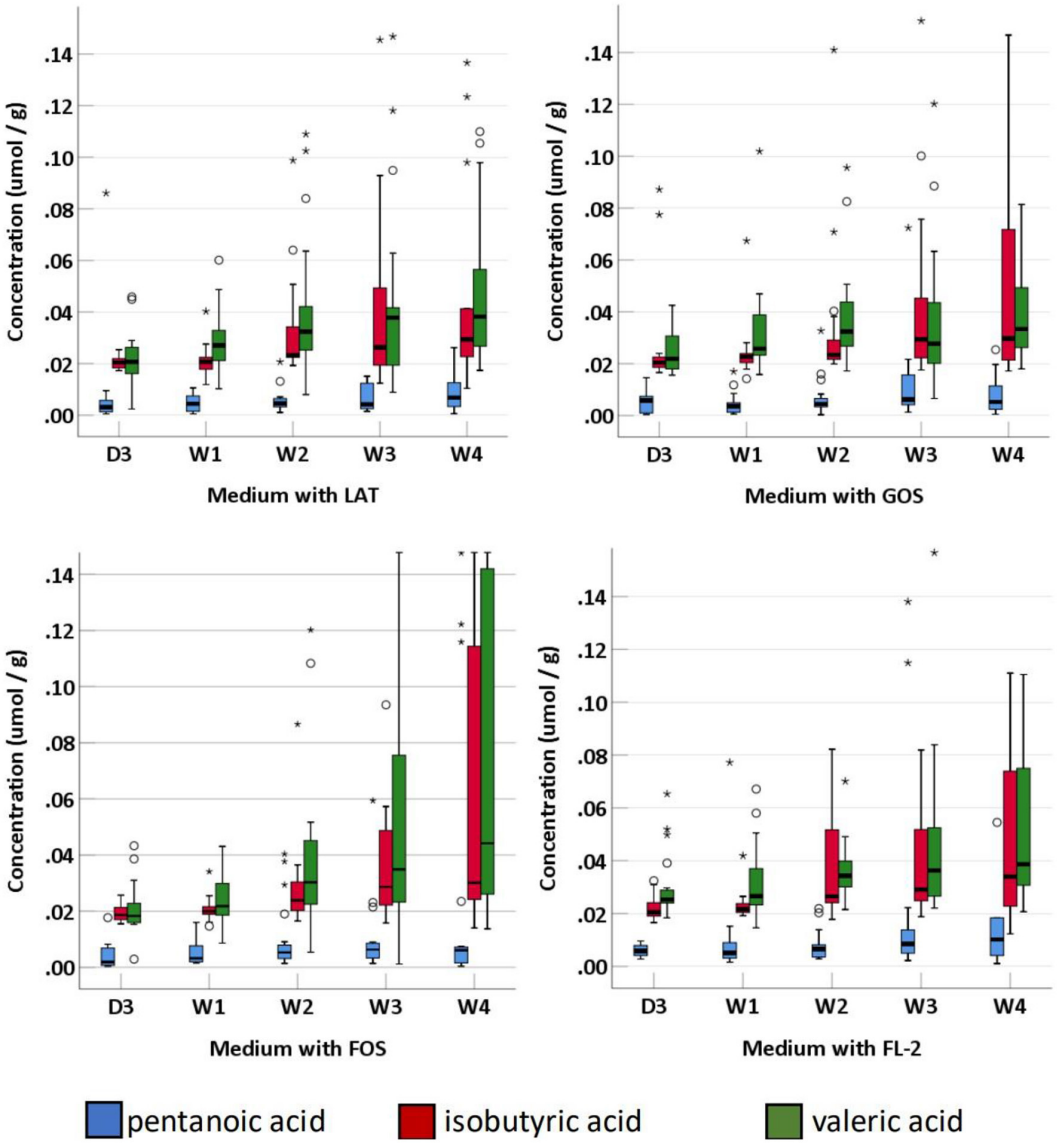
The concentration of fecal SCFAs (pentanoic acid, isobutyric acid, and valeric acid) of preterm infants of different ages. D3, 3 days after birth; W1, 1 week after birth; W2, 2 weeks after birth; W3, 3 weeks after birth; W4, 4 weeks after birth; LAT, lactose; FOS, fructooligosaccharide; FL-2, milk oligosaccharide; GOS, galactooligosaccharide; ^*^, extreme value; °, discrete value; SCFAs, short-chain fatty acids.

**Figure 5:**
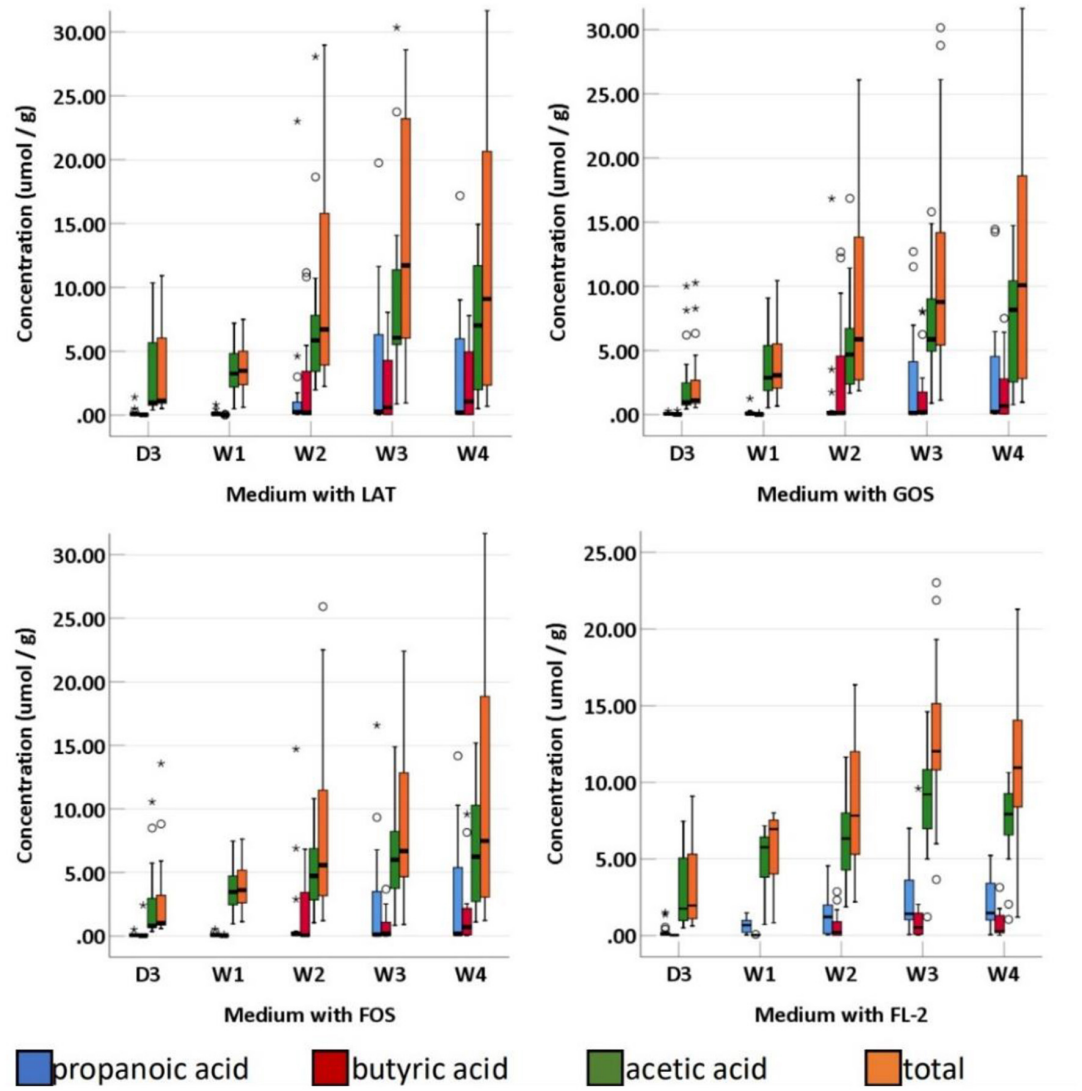
The concentration of fecal SCFAs (propanoic acid, butyric acid, acetic acid, and total) of preterm infants of different ages. D3, 3 days after birth; W1, 1 Week after birth; W2, 2 weeks after birth; W3, 3 weeks after birth; W4, 4 weeks after birth; LAT, lactose; FOS, fructooligosaccharide; FL-2, milk oligosaccharide; GOS, galactooligosaccharide; ^*^, extreme value; °, discrete value; SCFAs, short-chain fatty acids.

SCFAs were measured in a total of 95 fecal specimens. In medium with LAT, the concentration of total SCFAs produced by preterm infant gut microbiota was 1.14 (0.88–7.09) μmol/g within D3; 3.46 (2.37–5.13) μmol/g at W_1_; 6.71 (3.73–17.08) μmol/g at W_2_; 11.71 (5.86–23.77) μmol/g at W3; and 9.09 (2.11–23.24) μmol/g at W4. In medium with GOS, the concentration of total SCFAs was 1.11 (0.89–2.93) μmol/g within D3; 3.07 (2.04–5.54) μmol/g at W_1_; 5.88 (2.68–15.34) μmol/g at W2; 8.78 (5.38–17.66) μmol/g at W3; and 10.09 (2.56–19.25) μmol/g at W4. In medium with FOS, the concentration of total SCFAs was 1.01 (0.86–3.65) μmol/g within D_3_; 3.62 (2.56–5.40) μmol/g at W1; 5.57 (2.98–11.94) μmol/g at W2; 6.68 (4.53–13.15) μmol/g at W3; and 7.49 (2.93–19.19) μmol/g at W4. In medium with FL-2, the concentration of total SCFAs was 1.96 (1.06–7.09) μmol/g within D3; 6.95 (3.86–7.57) μmol/g at W1; 7.82 (4.38–12.75) μmol/g at W2; 12.03 (10.31–15.57) μmol/g at W3; and 10.93 (8.39–14.36) μmol/g at W4. The concentrations of total SCFAs, acetic acid, propanoic acid, butyric acid, isobutyric acid, pentanoic acid, and valeric acid in the four media usually increased with age (p-trends < 0.05) (Figures 4, 5). Among them, acetic acid was the predominant SCFA, and the concentration of pentanoic acid was the lowest. When stratified by birth weight (<1,500 and ≥1,500 g), gender, or delivery mode, differences in the concentrations of SCFAs were not significant (*p*-trends > 0.05).

## Discussion

We followed the development of the intestinal gases and SCFAs produced by gut microbiota *in vitro* longitudinally in a cohort of 19 prematurely born infants from birth to 4 weeks. We examined the change in the composition and volume of intestinal gases and SCFA profiles produced by preterm infant gut microbiota *in vitro* during the first 4 weeks of life. We found that the intestinal gases and SCFAs produced by gut microbiota *in vitro* increased with age during the first 4 weeks of life. A number of H_2_ and CO_2_ began to be detected at 2 weeks. This change has a strong association with gut microbiota colonization, which develops over the course of host infancy to eventually reach its adult form (17, 18). The gut microbiota community occurs during the first 3 years of life. As the diversity and number of gut microbiota increase in the first several years, the volume and composition of intestinal gases change constantly.

CO_2_ can be produced after each food intake in the stomach or in the rest of the gut segments. CO_2_ is a major gas generated during the bacterial fermentation of carbohydrates in the distal small intestine and colon. Our results showed that CO_2_ was usually the predominant intestinal gas in preterm infants, which is consistent with previous studies (9). Additionally, we found that the concentration of CO_2_ increased sharply at 2 weeks after birth. This result indicated that the massive colonization of gut microbiota that produces CO_2_ may begin at 2 weeks after the birth of preterm infants.

Previous studies indicated that a broad assemblage of hydrogenogens must exist in the human colon. Most likely, it is most abundant in the right colon, the colonic region with the greatest extent of microbial fermentation (19). H_2_ production is integral to broad microbial fermentation. Our study found that the proportion of H_2_ produced by the gut microbiota within the 3-day and first-week fecal samples was almost zero. The time at which H_2_ began to be detected was 2 weeks after birth. A number of hydrogenogens may begin to colonize the gut of preterm infants at 2 weeks after birth. In addition, the concentration of H_2_ in the medium with LAT was greater than that in the other three media. According to a previous study, patients diagnosed with lactose (LAT) intolerance excreted more H_2_ *via* the lungs, which was detected by the LAT breath test (20, 21). It is apparent that the breath test is not suitable for neonates. The fermentation *in vitro* and gas analysis system may therefore be a useful tool for the diagnosis of LAT intolerance in neonates.

Peled et al. analyzed CH_4_ concentration in the expired air of 393 infants, children, and adolescents and found that there was no CH_4_ production below the age of 3 years (22). However, Methanobacteria were detected in fecal samples obtained from children at 27 months of age (23). Dridi's study indicated a high prevalence of *Methanobrevibacter smithii* and *Methanosphaera stadtmanae* in the human gut using an improved DNA detection protocol, and the youngest subject in this study was 1 month (24). In our study, CH_4_ was detected in the majority of samples. This result may prove that neonates may produce CH_4_, but only when the production reaches a threshold does it appear in the breath (25). In addition, we found that there was no difference in the proportion of CH_4_ among the groups classified by age. Previous studies showed that the quantities of *M. smithii* in human feces remained constant over time. Methanogenic activity seems to have remained unaltered over a period of many years with dietary and antibiotic changes (26). This trend of stability of *M. smithii* has also been reported over a 13-month period using fecal specimens from two adult individuals (27). Those studies can at least partly explain our results.

H_2_S is a notoriously toxic gas. Despite its lethal effects, H_2_S is a vital molecule for living organisms, including humans. It is involved in inflammation, gut motility, oxidative stress, ulcer healing, vascular tone, neuromodulation, cryoprotection, memory formation, hormone secretion, apoptosis, and many other vital biological functions (28). The concept was proposed decades before that H_2_S may increase the risk of mucosal inflammation (29). Recently, an increasing number of studies have suggested that a limited amount of H_2_S is necessary for reducing the risk of colonic mucosal inflammation (30–32). Our results showed that H_2_S was detectable in a small portion of samples. Among the preterm infants in our study, a high concentration of H_2_S was detected in the fecal samples of an infant diagnosed with neonatal necrotizing enterocolitis (NEC). Besides the intestinal gases, fecal volatile organic compounds (VOCs) are other products of metabolic pathways of intestinal microorganisms. Recently, more and more studies showed the potential of VOC analysis as an early diagnostic biomarker for NEC in preterm infants (33–35). Therefore, we hypothesized that the gas analyzer can be used as a new tool for exploring the relationship between H_2_S and NEC, which needs a large number of experiments to confirm.

A recent study has showed that VOC profiles, as measured by an eNose device, in preterm infants born at GA <30 weeks, are not influenced by GA or mode of delivery during the first 3 weeks of life (36). In this study, we evaluated the effects of mode of delivery, birth weight, gender, and type of feeding on intestinal gases. When stratified by birth weight (<1,500 and ≥1,500 g), only the concentration of CO_2_ was different between the two groups. When stratified by gender, type of feeding, or delivery mode, differences in the volume of total intestinal gases and the concentrations of CO_2_, H_2_, CH_4_, and H_2_S were not significant between the two groups. This result indicates that birth weight may influence the production of CO_2_ in the human gut. However, gender and delivery mode have little impact on intestinal gas production. More samples are needed to confirm our results.

SCFAs are the major end products of bacterial metabolism in the human large intestine. The principal SCFAs that result from both carbohydrate and amino acid fermentation are acetate, propionate, and butyrate (37), which are similar to the SCFA profiles of preterm infants. In addition, we found that the concentrations of total SCFAs, acetic acid, propanoic acid, butyric acid, isobutyric acid, pentanoic acid, and valeric acid in the four media usually increased with age. Previous findings showed that SCFA formation by intestinal bacteria was regulated by many different host, environmental, dietary, and microbiological factors (37). In the past decade, several studies have indicated that feeding types and perinatal antibiotics significantly affect the function of the gut microbiota and the content of SCFAs in the stool samples of premature infants (38–40). When stratified by birth weight (<1,500 and ≥1,500 g), gender, or delivery mode, differences in the concentrations of SCFAs in preterm infants were not significant. More samples and grouping are needed to confirm our findings. SCFAs represent the major carbon flux from the diet through the gut microbiota to the host, and evidence is emerging for a regulatory role of SCFAs in local, intermediary, and peripheral metabolism. Manipulating the diet-gut microbiome–host metabolism axis may be a useful prevention of some leading causes of morbidity and mortality (4). However, our understanding of the significance of SCFAs in human metabolic health is limited, especially in neonates. Thus, more well-designed and controlled human studies are needed.

The first strength of this study was the prospective and standardized collection and handling of fecal samples by which we reduce the risk of potential pre-analytical errors. Secondly, we excluded the measurement of disease-specific intestinal gases and SCFAs by excluding infants with obvious malformations or inherited or metabolic diseases. Lastly, the gas analyzer (HL-QT01) used in this study was noninvasive compared with the tonometric balloon technique and intestinal washout technique used in other studies (6, 41).

There are several limitations that need to be addressed. Firstly, the study sample size was relatively small to evaluate the effects of delivery mode, birth weight, gender, and feeding type on intestinal gases and SCFAs concentrations among these heterogeneous premature babies. Large studies are needed to confirm our findings. Secondly, there was no microbiome analysis. Although we did not directly conduct gut microbiome analysis, in *vitro* fermentation methodology was a sophisticated platform for studying the gut microbiota composition and functionality (42).

Lastly, this was an *in vitro* study. In a variety of studies, researchers have used *in vitro* fermentations to explore fermentation rate, SCFA production, and changes in microbiota in existence of dietary fibers (43). Batch *in vitro* fermentations, which were generally carried out in test tubes within a short period of time (usually 24–48 h), was the most convenient way for studying microbial degradation of specific dietary compounds and therefore to explore the metabolites released and how the host health could be affected (42). LAT, FOS, GOS, and FL-2 were generally thought to be fermented in the colon of infants and degraded into physiologically active metabolites and gases. Therefore, the capability of infant gut microbiota degrading oligosaccharides (including LAT, FOS, GOS, and FL-2) to produce intestinal gases and SCFAs was compared in our study. We found that *in vitro* batch fermentation could be further used as a valuable screening platform for initial examination of food stuffs or food components and could help optimize individualized nutrition in infants.

As *in vitro* models do not involve epithelial or immune cells, it is true that their applicability to study complexity of the human colon and host–gut microbiota interactions are limited. However, *in vitro* models have their own advantages. Firstly, they offer unique opportunities for quantitative measurements of particular microbial metabolites. As the majority of SCFAs are absorbed by colonic epithelial cells and metabolized by cells in different tissues including colon cells, only 5%−10% are excreted in the feces (43). Measurement of fecal SCFAs may lead to overestimation or underestimation of intestinal microbial metabolism. Secondly, *in vitro* models also provide tools to monitor the metabolic activities of the microbiota, rather than merely description of fecal bacterial composition or metabolite composition.

In summary, our study demonstrated that the intestinal gases and SCFAs produced by preterm infant gut microbiota *in vitro* increased with age during the first 4 weeks of life. The production of intestinal gases and SCFAs is strongly associated with gut microbiota colonization and health status. More well-designed and controlled human studies are needed to explore the associations between intestinal gases and SCFA production with gut microbiota colonization and diseases of preterm infants.

## Data Availability Statement

The original contributions presented in the study are included in the article/Supplementary Materials, further inquiries can be directed to the corresponding author/s.

## Ethics Statement

The studies involving human participants were reviewed and approved by Ethics Committee of Shanghai Children's Hospital. Written informed consent to participate in this study was provided by the participants' legal guardian/next of kin. Written informed consent was obtained from the minor(s)' legal guardian/next of kin for the publication of any potentially identifiable images or data included in this article.

## Author Contributions

YH and GD: conceptualization and methodology. XW and JL: formal analysis and writing—original draft preparation. DY and HZ: collected clinical samples and performed experiments. NL and KG: data curation. YH: writing—review and editing and funding acquisition. All authors contributed to the article and approved the submitted version.

## Funding

This work was supported by the Special Project of Clinical Research on Health Industry of Shanghai Health Commission (Grant Number 201940329) and Special Project of Clinical Research of Shanghai Children's Hospital (Grant Number 2020YLYZ01).

## Conflict of Interest

The authors declare that the research was conducted in the absence of any commercial or financial relationships that could be construed as a potential conflict of interest.

## Publisher's Note

All claims expressed in this article are solely those of the authors and do not necessarily represent those of their affiliated organizations, or those of the publisher, the editors and the reviewers. Any product that may be evaluated in this article, or claim that may be made by its manufacturer, is not guaranteed or endorsed by the publisher.

## Abbreviations

SCFAs: short-chain fatty acids
NICU: neonatal intensive care unit
LAT: lactose
FOS: fructooligosaccharide
FL-2: milk oligosaccharide
GOS: galactooligosaccharide
CO_2_: carbon dioxide
H_2_: hydrogen
CH_4_: methane
H_2_S: hydrogen sulfide
GC: gas chromatography
N_2_: nitrogen
SIBO: small intestinal bacterial over growth
CRC: colorectal cancer
AP: adenomatous polyposis
BMJ: breast milk jaundice
CMPA: cow's milk protein allergy
95% CI: 95% confidence intervals
IQR: interquartile range
D3: three days after birth
W1: 1 week after birth
W2: 2 weeks after birth
W3: 3 weeks after birth
W4: 4 weeks after birth
NEC: necrotizing enterocolitis
VOC: fecal volatile organic compound.
